# Factors associated with cutaneous colonization of Mucormycetes in diabetic and non-diabetic individuals

**DOI:** 10.1099/acmi.0.000495.v4

**Published:** 2023-09-06

**Authors:** Bhuvan Saud, Kajal Chand, Neetu Amatya, Govinda Paudel, Saroj Adhikari, Vikram Shrestha

**Affiliations:** ^1^​ Department of Medical Laboratory Technology, Janamaitri Foundation Institute of Health Sciences, Lalitpur, Nepal; ^2^​ Ministry of Health and Population, Government of Nepal, Kathmandu, Nepal

**Keywords:** amphotericin B, cutaneous colonization, diabetic, mucormycetes, *Mucor* species

## Abstract

This study was conducted to assess factors associated with cutaneous colonization of Mucormycetes in diabetic and non-diabetic individuals. A total of 800 swab samples from 200 participants including 100 diabetics and 100 non-diabetics were collected from four different body sites: (1) the forehead, (2) nasal cavity, (3) hands and (4) feet. Fungal isolation, fungal identification and antibiotic sensitivity tests were performed on the isolates. Overall, 12.0 % of the participants showed Mucormycetes colonization while the commonest fungal isolates were *Mucor* Species (Spp.). followed by *Rhizopus* spp. Diabetics had a 11 times higher odds of colonization compared to non-diabetics. The majority of the isolates were resistant to itraconazole; however, all isolates were sensitive to amphotericin B. A significant association was observed between profession and Mucormycetes (*P*=0.03) with significantly higher colonization in retired people compared to business people. Higher odds of colonization were demonstrated among older ages, lower class status and individuals with prolonged contact time with soil.

## Data Summary

Supplementary information can be found in Figshare (https://doi.org/10.6084/m9.figshare.21091273.v2) [1].

## Introduction

Admist recent Coronavirus Disease 2019 (COVID-19) pandemic, the public became more aware of mucormycosis, but this is not a new disease. Mucormycetes are ubiquitous and spread via inhalation and inoculation [2]. Mucormycetes cause a serious but rare fungal infection called mucormycosis (previously called sygomycosis) [3]. These fungi cause mild to life-threatening disseminated infections depending upon the host’s immune condition, site of colonization and variant of the fungus [2]. Conditions such as malignancies, diabetes, trauma, burns, transplantation, malnutrition, immunosuppressive conditions and therapies are associated with a high risk of infection [4, 5]. Reported mortality rates in disseminated, pulmonary and sinus infections are 96.0, 76.0 and 46.0 %, respectively [6].

Diabetes is an endocrine condition in which blood glucose levels become elevated, which results in impaired immunological function that offers an ideal environment for fungal growth [7, 8]. Globally, the prevalence of diabetes has been rising rapidly, especially in developing countries. In Nepal, the prevalence of pre-diabetics and diabetics is 19.5 and 9.5 %, respectively, with roughly 15.0 % of diabetic individuals living in metropolitan areas [9]. Nepal is lacking adequate facilities for fungal infection diagnostics and lacks optimum understanding regarding fungal diseases. This means that diabetics are missing prompt diagnosis of any possible fungal infection. Mucormycosis is a rare fungal disease among diabetics, but it causes life-threatening complications if not treated in a timely manner [10]. Social factors such as socio-economic status, occupation, age and level of education level can contribute to colonization of these fungi among diabetics [2]. Research on Mucormycetes has mainly focused on mechanisms involved in infection, and there are limited studies regarding its asymptomatic colonization among immune compromised individuals. The present study was designed to investigate the status of asymptomatic colonization of Mucormycetes in diabetic and non-diabetic individuals. It is the first study in Nepal to investigate the role of social factors in fungal colonization.

## Methods

### Study design and sample collection

A case-control study was conducted in Kathmandu Metropolitan area, Nepal, between January and July 2022. A total of 200 participants (100 diabetic and 100 non-diabetic), aged 18 years and above, were enrolled. Swab samples were collected from five different sites such as the forehead, nasal cavity, hands and feet. Samples were then transported to the Department of Medical Laboratory Technology within 1 h, maintaining a cold chain. Participants who were on antifungal medication and had symptomatic fungal infection during the sampling period were excluded.

### Fungus isolation, identification and antifungal testing

Swab samples were inoculated on Sabouraud Dextrose Agar (Hi-Media) containing chloramphenicol at 50 mg l^−1^ and were incubated aerobically for 3–5 days at room temperature (25–30 °C). Identification of Mucormycetes was carried out based on colony morphology and microscopic examination, using the scotch tape method and the tease mount preparation method. Isolates were transferred to Sabouraud Dextrose Broth (Hi-Media) to obtain turbidity equivalent to McFarland standard 0.5. The preparations were then inoculated on Mueller Hinton Agar (Hi-Media) plates using a lawn culture technique for antifungal susceptibility testing. Interpretation of results was carried out following guidelines of the Clinical and Laboratory Standards Institute (CLSI) [11]. Commercially available antifungal discs used were amphotericin B (10 µg; Hi-Media), voriconazole (1 µg; Hi-Media) and itraconazole (1 µg; Hi-Media). *Candida krusei* (ATCC 6258) and *Aspergillus flavus* (ATCC 204304) were used as quality control strains.

### Data analysis

The obtained data were entered into and analysed using Statistical Package for the Social Sciences (IBM) version 21. Descriptive and analytical statistics were performed. Fischer’s exact test was used to determine the association between independent variables and fungal colonization among both groups considering a *P*-value of <0.05 as statistically significant.

## Results and discussion

Mucormycosis is a rare fungal disease with a high mortality rate. The number of cases in 2010 was found to have increased more than 5.5-fold when compared with cases in 1990 [12]. The disease is often associated with underlying health conditions such as diabetes, organ transplantation, immunosuppressive status and cancer. Annually, the incidence of mucormycosis is about 1.7 cases per 1 000 000 individuals [13]. During the recent Severe Acute Respiratory Syndrome Coronavirus-2 (SARS-CoV-2) pandemic, mucormycosis was mainly reported in patients with uncontrolled hyperglycaemic conditions [14]. This association can be explained by the fact that an uncontrolled diabetic condition leads to dysfunction of the immune system, which leaves patients more susceptible to colonization by fungi such as Mucormycetes. The prevalence of diabetic individuals in low- and middle-income countries is higher and rapidly increasing in comparison with that in high-income countries [15, 16].

Mucormycetes were found in 12.0 % (24/200) of the participants, 22 of whom were diabetics and two were non-diabetics. Therefore, the rate of fungus colonization in diabetics is 11 times higher than that in non-diabetics. In diabetics, *Mucor* spp. was the predominant fungus, followed by *Rhizopus* spp., *Rhizomucor* spp. and *Syncephalastrum* spp., respectively. On the other hand, in non-diabetics, only *Mucor* spp. was isolated (Table 1). Our previous study also found that only diabetics were positive for *Mucor* spp. and *Rhizopus* spp. colonization [17]. A retrospective study that analysed 6 years of data on Mucormycetes in Australia found that 32.0 % of cases had invasive fungal disease and 56.0 % were colonized mainly by *Rhizopus* spp., *Rhizomucor* spp. and *Mucor* spp. [18]. Another prospective study found 388 proven/probable Mucormycosis cases, of which *Rhizopus arrhizus*, *Rhizopus microspores*, *Apophysomyces variabilis* and *Rhizopus homothallicus* were reported in descending order of prevalence among uncontrolled diabetics [19]. Similarly, another study noted that *Rhizopus oryzae* was the main organism in patients with diabetes [6]. Diabetics have diminished chemotaxis and phagocytosis activity, increased iron availability and a lower pH that facilitate mucormycosis development [20]. This leads to higher vulnerability to severe complications related to mucormycosis. Common clinical conditions include cavernous sinus thrombosis, disseminated infection, periorbital destruction, palatine ulcers and osteomyelitis that can lead to death of the patient if not treated early and appropriately [4, 9].

**Table 1.** Distribution of fungal isolates among study individuals and its association with variables

Variable	Distribution			Positive cases		OR (95 % CI)	*P*-value†
	Overall values (*N*=200), *n* (%)	D (*N*=100)	ND (*N*=100)	D (*N*=22), *n* (%)	ND (*N*=2), *n* (%)		
**Gender**							
Male	81 (40.5)	49	32	9 (40.9)	0	Ref.	0.51
Female	119 (59.5)	51	68	13 (59.1)	2 (100.0)	0.28 (0.01–6.62)	
**Age interval (years)**							
<30	5 (2.5)	0	5	0	0 (0 %)	Ref.	0.38
31–40	70 (35.0)	9	51	4 (18.2)	0 (0 %)	9.0 (0.07–1195.50)	
41–50	45 (22.5)	16	29	6 (27.3)	1 (50 %)	4.3 (0.06–320.42)	
51–60	38 (19.0)	33	5	3 (13.6)	1 (50 %)	2.3 (0.03–182.92)	
>60	42 (21.0)	42	10	9 (40.8)	0	19.0 (0.15–2409.97)	
**Level of education**							
Uneducated	39 (19.5)	38	1	13 (59.0)	1(50.0)	Ref.	0.67
Primary	16 (8.0)	15	1	4 (18.2)	1(50.0)	0.31 (0.02–6.12)	
Secondary	13 (6.5)	12	1	2 (9.0)	0	0.56 (0.02–17.92)	
SLC	13 (6.5)	11	2	2 (9.0)	0	0.56 (0.02–17.92)	
Higher secondary	25 (12.5)	13	12	1 (4.5)	0	0.3 (0.01–12.42)	
Bachelors degree	85 (42.5)	9	76	0	0	0.1 (0.001–7.93)	
Masters degree	9 (4.5)	2	7	0	0	0.1 (0.001–7.93)	
**Profession**							
Business	22 (11.0)	15	7	0	1 (50.0)	Ref.	0.03*
Services	82 (41.0)	7	75	3 (13.6)	1 (50.0)	7.0 (0.17–291.36)	
Retired	61 (30.5)	59	2	15 (61.2)	0	93.0 (1.31–6606.77)	
Other	35 (17.5)	19	16	4 (18.1)	0	27.0 (0.35–2058.114)	
**Socioeconomic status**							
Upper	1 (0.5)	0	1	0	0	Ref.	0.42
Upper middle	3 (1.5)	1	2	1 (4.5)	0	3.0 (0.02–473.10)	
Lower middle	166 (83.0)	81	85	3 (13.6)	1(50.0)	2.3 (0.03–182.92)	
Upper lower	23 (11.5)	13	10	13 (59.0)	1(50.0)	9.0 (0.13–642.12)	
Lower	7 (3.5)	5	2	5 (22.7)	0	11.0 (0.08–1438.12)	
**Contact with soil**							
Low	97 (48.5)	25	72	3 (13.6)	0	Ref.	0.37
Medium	78 (39.0)	54	24	7 (31.8)	2 (100.0)	0.43 (0.02–11.51)	
High	25 (12.5)	21	4	12 (54.5)	0	3.57 (0.06–214.49)	
**Site of isolation**							
Forehead	–	–	–	5 (22.7)	0	Ref.	0.83
Feet	–	–	–	7 (31.8)	1 (50.0)	0.45 (0.02–13.41)	
Hands	–	–	–	4 (18.2)	1 (50.0)	0.27 (0.01–8.46)	
Nasal cavity	–	–	–	6 (27.2)	0	1.18 (0.02–69.98)	

D, diabetic; ND, non-diabetic; *n*, number; OR odds ratio; Ref., reference; SLC, School Leaving Certificate.

*Significant at *P*<0.05.

†Fischer’s exact test.

We found that the colonization of Mucormycetes was higher in female participants. Interestingly, among non-diabetic participants, only females were positive for colonization. In addition, colonization is often associated with socio-economic status, profession and contact with soil. We noted that colonization decreases with an increase in level of education: uneducated participants showed the highest colonization, followed by those of primary, secondary and school leaving certificate education. Furthermore, retired participants had a higher colonization rate than working participants. Here, the elderly age group (>60 years) seemed to have a 19 times greater risk of Mucormycetes colonization than adults. A statistically significant relationship (*P*=0.03) was observed between participants’ profession and fungal isolation, where retired people were found to be at higher risk of fungal colonization. It has previously been observed that immunosenescence starts in old age, and pathogens can easily colonize and establish infection [21]. The immune system gradually weakens with age, conferring decreased immune cell production (B-cells and T-cells) and function, allowing pathogens to colonize easily and cause illness [22].

In addition, those who frequently come into contact with soil had a three times higher odds of colonization than those with less contact. Mucorales are present throughout the environment, growing in organic substances such as food, dead plants and animal waste materials. Therefore, spores of these pathogens can enter the body through inhalation and skin contact. This may also cause cutaneous mucormycosis without underlying conditions. We have found that colonization mainly occurred in the feet, followed by the nasal cavity, forehead and hands, indicating inadequate hygiene among people working in the fields. A recent study found that 22.2 % of cases of asymptomatic airway colonization were due to Mucorales [23]. The fungi are transmitted mainly via inhalation of spores, which are further deposited in the paranasal sinuses. Germination and establishment of fungi are favoured by the moist surface of the nasal cavity [24]. Similarly, individuals of lower and upper lower socio-economic status were relatively more prone to colonization than those with higher incomes. A lack of health education regarding diabetes and mucormycosis, inadequate sanitization, and low socio-economic status can contribute to mucormycosis among diabetics.

With regard to drug resistance, *Mucor* spp*.* were resistant to itraconazole (14, 87.5 %) and voriconazole (11, 68.8 %). All four *Rhizopus* spp*.* isolates were resistant to itraconazole (4, 100.0 %), while three were resistant to voriconazole (3, 75.0 %). In addition, *Rhizomucor* spp*.* (1, 50.0 %) and *Syncephalastrum* spp*.* (2, 100.0 %) showed resistance to itraconazole, whilst *Syncephalastrum* isolates were sensitive to voriconazole. In addition, all 24 isolates were sensitive to amphotericin B (Table 2). Detailed information related to this research is included in the supplementary material file [1]. During the COVID-19 pandemic, amphotericin B, itraconazole and other antifungal drugs were used to treat mucormycosis co-infection [20]. A study found that *Mucor* spp. and *Rhizopus* spp. isolates from environmental samples were resistant to voriconazole and itraconazole [25].

**Table 2.** Antifungal resistance pattern of clinical isolates

Fungal isolates (*n*)	Itraconazole, *n* (%)	Voriconazole, *n* (%)	Amphotericin-B, *n* (%)
*Mucor* spp. (16)	14 (87.5)	11 (68.8)	0
*Rhizopus* spp. (4)	4 (100.0)	3 (75.0)	0
*Rhizomucor* spp. (2)	1 (50.0)	0	0
*Syncephalastrum* spp. (2)	2 (100.0)	0	0

## Conclusion

People with diabetes are highly prone to colonization by cutaneous mycoses. A logistic regression analysis concluded that soil contact, old age and lower socio-economic status contributed to colonization. It is necessary to promote hygiene knowledge and awareness among diabetics to prevent any future serious complications from such infection. Likewise, a continuity of care programme for diabetics can help prevent uncontrolled diabetes and improve quality of life, which can further contribute to preventing the dissemination of mucormycosis infection.
